# Protective Effect of Multifloral Honey on Stem Cell Aging in a Dynamic Cell Culture Model

**DOI:** 10.3390/antiox15010115

**Published:** 2026-01-16

**Authors:** Fikriye Fulya Kavak, Sara Cruciani, Giuseppe Garroni, Diletta Serra, Rosanna Satta, Ibrahim Pirim, Melek Pehlivan, Margherita Maioli

**Affiliations:** 1 Department of Biomedical Sciences, University of Sassari, Viale San Pietro 43/B, 07100 Sassari, Italy; fikriyefulyakavak@gmail.com (F.F.K.); scruciani@uniss.it (S.C.); giugarroni21@gmail.com (G.G.); dilettaserra9@gmail.com (D.S.); 2 Department of Medicine, Surgery and Pharmacy, University of Sassari, 07100 Sassari, Italy; rsatta@uniss.it; 3 Department of Medical Biology, Faculty of Medicine, Izmir Katip Celebi University, Izmir 35620, Türkiye; ibrahim.pirim@gmail.com; 4 Department of Medical Laboratory Techniques, Vocational School of Health Services, Izmir Katip Celebi University, Izmir 35620, Türkiye

**Keywords:** stem cells, aging, bioactive molecules, regenerative medicine

## Abstract

Natural compounds, as honey-derived flavonoids and phenolic compounds, are increasingly investigated for their potential to mitigate skin aging and prevent oxidative stress-induced cellular damages. In this context, a dynamic cell culture model was employed to assess the protective influence of honey pre-treatment on stem cell–associated genes and the Wingless-related integration site (Wnt) signaling pathway following ultraviolet (UV)-induced aging. Using a bioreactor, skin stem cells (SSCs) derived from healthy skin biopsies and human skin fibroblasts (HFF1) were pre-treated with 1% honey for 48 h and then exposed to UV. Real-time quantitative polymerase chain reaction (RT-qPCR) analyses were performed on Wnt signaling and anti-aging molecular responses. Honey pre-treatment enhanced the expression of pluripotency markers (Octamer-binding transcription factor 4 (Oct4); SRY-box transcription factor 2 (Sox2)) and reduced senescence-related cell cycle regulators (cyclin-dependent kinase inhibitor 2A (p16); cyclin-dependent kinase inhibitor 1A (p21); tumor protein 53 (p53)) in SSCs. In UV-damaged SSCs, honey also significantly increased Wnt3a expression. In fibroblasts, honey pre-treatment upregulated Heat shock protein 70 (Hsp70) and Hyaluronan synthase 2 (HAS2) expression, while downregulating caspase-8 (CASP8), indicating a protective role against UV-mediated cellular stress. We also analyzed nitric oxide release and the total antioxidant capacity of cells after treatment. Collectively, these findings suggest that honey may safeguard skin stem cells from UV-induced aging by modulating pluripotency and senescence-associated genes and regulating differentiation through alterations in Wnt signaling. Furthermore, Hsp70 upregulation in fibroblasts appears to strengthen cellular stress responses and support homeostatic stability.

## 1. Introduction

The skin, as the largest organ of the human body, acts as the first line of defense against external environmental factors, with ultraviolet radiation being one of the most detrimental [1]. UV exposure, particularly UVB and UVC, leads to significant damages at the cellular level, accelerating the skin aging process through DNA damage, oxidative stress, and reduced cellular renewal capabilities [2]. This damages not only manifests aesthetically, in the form of wrinkles and pigmentation, but also increases the risk of skin cancers and other disorders. Mesenchymal stem cells (MSCs) are pivotal in maintaining skin homeostasis due to their ability to differentiate and replace damaged cells [2,3]. A decreased number or activity of stem cells, which are essential for maintaining skin stability, is associated with premature aging and skin cancer. UV-induced stress can lead to premature senescence of these cells, decreasing their regenerative potential and impacting overall skin health [4]. Recent research highlights the importance of protecting MSCs from such damages to maintain their pluripotency and ensure effective tissue regeneration.

Honey has been used for medicinal purposes for over 2000 years. It contains bioactive components such as sugars, water, proteins, vitamins, minerals, and natural antioxidant compounds, including phenolic compounds and flavonoids [5,6]. Previous studies showed that honey-derived phytochemicals can modulate fundamental molecular pathways of aging. Compounds as acacetin, pinocembrin, chrysin, syringic acid, and caffeic acid have been reported to activate stem cell–related signals (*Oct4/Sox2, Wnt/beta-catenin (β-catenin)*), stimulate longevity-associated enzymes (sirtuins), and alleviate oxidative stress and DNA damage [7,8,9,10,11]. Moreover, honey possesses therapeutic potential in treating several diseases through oxidative stress, inflammation and apoptosis and may also play a role on skin regeneration and cancer [6]. In addition, honey has been reported to support tissue homeostasis by preserving stem cell viability and can be a useful biomaterial in stem cell-based therapies. Moreover, its positive effects on fat and umbilical cord-derived stem cells have been identified [12,13]. Oryan et al. colleagues showed in a rat burn model that the combination of adipose-derived stem cells and honey decreased pro-inflammatory cytokines (Interleukin 1 Beta (IL-1β); Transforming Growth Factor Beta 1 (TGF-β1), increased basic fibroblast growth factor (bFGF) expression, boosted angiogenesis, re-epithelialization, and granulation tissue development. Higher bFGF levels at day 28 showed anti-scarring effects and revealed the capability of honey to provide adipose-derived stem cells viability support, enhancing resistance to oxidative stress and their regenerative capability [12]. Das et al. indicated that honey, owing to its antioxidant and anti-inflammatory roles, decreased reactive oxygen species and corresponding senescence indicators in umbilical cord-derived mesenchymal stem cells. Their studies revealed greater cell proliferation and lower beta-galactosidase (β-gal) positive senescence rates in cells grown on poly-vinyl alcohol (PVA):honey matrices than on pure PVA [13]. In addition to these cellular effects, honey may also affect intracellular signaling pathways that shape stem cell behavior. One of the most significant of these is the Wnt signaling pathway, which plays a central role in controlling stem cell proliferation, maintaining homeostasis, and guiding differentiation, and could therefore be one of the mechanisms through which honey supports regenerative processes [14,15,16]. Other Authors indicates that Wnt3a can enhance stem cell survival and proliferation, while the Wnt/β-catenin pathway modulates stem cell aging by influencing reactive oxidative species (ROS) production [17,18,19]. Furthermore, non-canonical Wnt signaling plays a fundamental regulatory role in processes as directed migration, cell polarity and microenvironmental sensitivity of skin cells [20]. Among other pathways, also Hsp70 and B lymphoma Mo-MLV insertion region 1 (Bmi1) are critical for adult stem cell protection and cancer regulation [21], inhibiting genes eliciting senescence and apoptosis [21,22], and inducing DNA repair and maintains extracellular matrix (ECM) integrity, potentially reducing wrinkle formation [23].

In this context, this study investigates the protective effects of multifloral honey from Eastern Anatolia against UV-induced aging in skin stem cells. Using a bioreactor that mimics the skin environment, we evaluate the impact of honey on stem cell maintenance, ECM production, and cellular aging mechanisms. Specifically, we analyze key genes involved in stemness and self-renewal, as Oct4 and Sox2, as well as genes related to the Wnt signaling pathway (Wnt3a, Wnt5a), associated with cell proliferation and differentiation. We also assessed the antioxidants properties of honey, measuring nitric oxide (NO) production and the total antioxidant capacity (TAC) of cells. The aim of this study was to explore the potential of natural compounds in preserving skin health and mitigating aging, contributing to the development of innovative therapeutic approaches.

## 2. Materials and Methods

### 2.1. Stem Cell Isolation, Culturing and Preparation of Other Cell Types

Human skin stem cells (SSCs) were obtained from biopsies of adult male and female patients following approval from the ethics commission (Ethical Clearance prot.n. 0059862, 21/09/2017-Commissione Etica CNR). The collected samples were mechanically isolated and cultured following a previously published protocol [24]. Human skin fibroblast 1 (HFF1) and Human skin keratinocytes (HaCaT) were acquired from ATCC (Manassas, VA, USA) and cultured in a Dulbecco’s modified Eagle’s Medium (DMEM) low-glucose medium (Life Technologies, Carlsbad, CA, USA), enriched with 10% fetal bovine serum (FBS) (Life Technologies, Carlsbad, CA, USA), 2 mM L-glutamine (Euroclone, Milano, Italy), and 1% penicillin/streptomycin (Euroclone, Milano, Italy) [25].

### 2.2. Preparation for Honey

Honey solutions were prepared by dissolving honey in DMEM to achieve a 10% *w*/*v* concentration. 1 g of honey was weighed on a precision scale and dissolved in 10 mL of DMEM in a 15 mL sterile falcon tube. Prior to each experiment, honey solutions at different concentrations were prepared from the main stock solution using the formula M1 × V1 = M2 × V2.

### 2.3. Analysis of Phytochemical Compounds UHPLC-Orbitrap^®^-HRMS

Raw honey was harvested in Autumn 2022 directly from beekeepers in the Eastern Anatolia region of Turkey. Honey has been stored at room temperature (20–25 °C), ensuring it remains protected from light. Phytochemical profiling was carried out using UHPLC-Orbitrap^®^ HRMS (Thermo Fisher Scientific, Waltham, MA, USA) in both positive and negative ionization modes. Chromatographic separation took place on a 3C18-EB (COSMOSIL, 100 × 2 mm) column with a methanol–water (0.5% acetic acid) gradient. Sample preparation and instrument settings followed the same protocol as described previously [26,27]. Analyses were performed by an external accredited laboratory service. Simultaneously, the physicochemical parameters and sugar profile were analyzed at the Ankara Food Control Laboratory Directorate (Report No: 2400415684/00), an ISO/IEC 17025 accredited facility. According to the official results, the honey exhibited a proline content of 943.8 mg/kg, which is significantly higher than the European (IHC) minimum requirement of 180 mg/kg [28] and Turkish Food Codex (300 mg/kg). The total invert sugar (fructose + glucose) was 71.5%, with a fructose-to-glucose ratio of 1.3. Additionally, the C4 sugar content was as low as 1.0%, and sucrose/maltose were not detected, confirming the high purity of the sample.

### 2.4. Cytotoxic Effect of Honey on HFF1 Cells and SSCs

Cytotoxicity analyses were performed using the MTT assay. HFF1 and SSCs were seeded in a 96-well plate with 5000 cells per well. The wells in the 96-well plates were grouped, and the cells in were treated for 24 and 48 h with honey solutions at concentrations ranging from 0.02% to 4% *w*/*v*. At the end of incubation period, honey solution was removed and 100 µL of MTT solution (5 mg/mL in PBS) were added to each well and incubated for 2 h at 37 °C. After incubation, the formazan crystals formed were dissolved by adding 100 µL of DMSO. The absorbance was measured at 570 nm using a microplate reader (Akribis Scientific, Common Farm, Frog Ln, Knutsford WA16 0JG, Great Britain). Based on the MTT results, the optimal time interval and concentration of honey were determined.

### 2.5. Setting up of Bioreactor and Cell Culture Conditions

Bioreactor is a technology that enables the separate cultivation of different cell types while assuring the mixing of cell environments through the continuous flow of fluid. To replicate the layers of skin, cells were cultivated in a dynamic flow bioreactor using chambers from Live Box2 (IVTech, Pisa, Italy).

**Figure 1:**
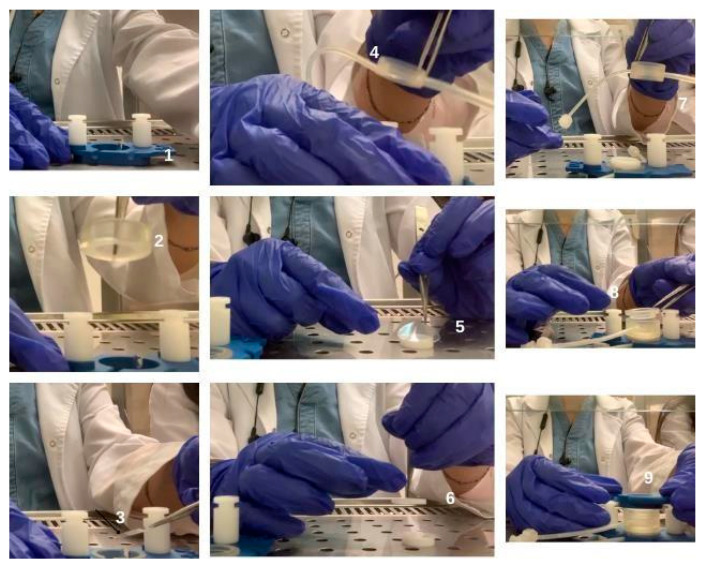
Setup of the bioreactor chamber.

**Figure 2:**
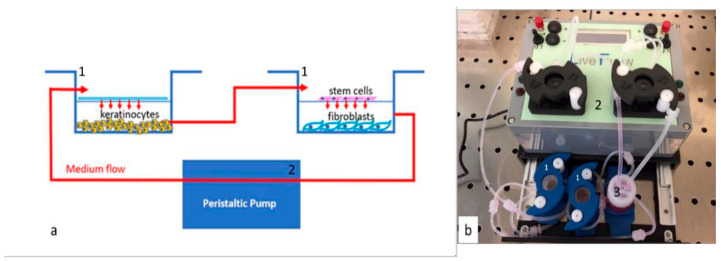
Schematic representation (**a**) and picture (**b**) of the bioreactor. Panel a represents the chambers (Live box2; blue lines (1)) and the cells cultured in each chamber. Red lines and arrows indicate the connections and medium flow to the chamber and pump (Live flow (2)). Panel b is a picture of the bioreactor in use (iVTech, italy). (1) Cell culture chamber (blue); (2) peristaltic pumps and (3) the reservoir for the culture medium [29].

Samples of each cell type were divided into groups as described in Table 1, and experiments in each group were performed at different times. Cells were counted with an automatic cell counter (LUNA, Logos biosystems, Gyeonggi-do, Republic of Korea) and seeded according to the isometric ratio of human skin tissue. 80,000 HaCaT, 40,000 HFF1 and 10,000 SSCs were planted in the chambers of the bioreactor (Figure 1). They were allowed to attach to the chambers for 24 h. At the end of 24 h, the chambers of the bioreactor were connected to each other, and the culture medium flowed through the connection pipes (Figure 2). Keratinocytes were used to establish a physiologically more relevant microenvironment within the dynamic culture system that more accurately mimics the in vivo cellular organization of the skin.

Cells were cultured in association with each other thanks to connecting chambers and a 0.45 µm membrane (ipCELLCULTURE™, it4ip, Ottignies-Louvain-la-Neuve, Belgium). The peristaltic pump [25] connects the chambers. After the flow rate of the culture medium was adjusted to 0.1 mL/min, the cells were left in culture for 48 h.

After the disassembly of the bioreactor setup, the chambers housing cells, both pre-treated (Uv + H) and untreated (UvC) with 1% honey for 48 h, were directly exposed to a UV lamp, with a wavelength ranging from 280 to 320 nm, positioned at 10 cm of distance for a duration of 2–3 min.

### 2.6. Gene Expression Analysis

For gene expression studies, RNA was extracted from each group of cells cultured in the above-described conditions. We then analyze the expression levels of stemness (Oct4, Sox2, Bmi1, TERT), cell cycle (p16, p21, p53), extracellular matrix (HAS2, Hsp70, CASP8), and Wnt signaling-related genes (Wnt3a, Wnt5a, Wnt7b, Wnt16, β-catenin, Dvl1, Dvl2, Dvl3, Axin1, Axin2, APC, GSK3β) in SSCs and HFF1 cells, providing insights into their roles in stem cell function, extracellular matrix regulation, and cellular aging (Table 2). Total mRNA was isolated from the pellets of the groups indicated in Table 1 (C, UvC, and H + Uv) using the RNeasy Mini Kit (Qiagen, Hilden, Germany) according to the manufacturer’s protocol. The amount and purity of RNA were measured by OD 260/280 nm using Nanodrop (Thermo Scientific, Waltham, MA USA). To detect gene expression levels, each sample were amplified in triplicate on a real-time PCR (RT-qPCR) device (Bio-Rad, Hercules, CA, USA) using the Luna One-Step RT-qPCR kit (Euroclone, Milan, Italy). The resulting Ct (cycle of threshold) values were normalized using GAPDH as a reference gene, and mRNA levels were expressed as the fold change (2^−∆∆Ct^) as compared to the untreated control (C).

### 2.7. Nitrite Quantification

To test the variation in NO production by the cells in the different culturing conditions, the Griess Reagent Kit for Nitrite Determination (Thermo Fisher Scientific, USA) was used. Briefly, 20 μL of Griess Reagent were added to 150 μL of nitrite containing samples in a 96-well plate and incubated for 30 min, according to the manufacturer’s protocol. The nitrite concentrations were read as the absorbance at 548 nm wavelength of the nitrite-containing samples in a spectrophotometric microplate reader (Akribis Scientific, Common Farm, Barrow, UK). Analysis was relative to the photometric reference sample.

### 2.8. Total Antioxidant Capacity

The measure of antioxidant proteins was performed using the Total Antioxidant Capacity Colorimetric Assay Kit (Abcam, Cambridge, UK). Cell culture media were collected for each sample cultured in the above-described conditions. According to the manufacturer’s protocol, 100 μL Cu^2+^ Working Solution were added to all standard and sample wells and incubated at room temperature for 90 min on an orbital shaker, protected from light. Absorbance was read using a microplate reader (Akribis Scientific, Common Farm, Great Britain) at OD 570 nm. Sample Total Antioxidant Capacity was calculated as: (Ts/Sv) * D, were Ts = TAC amount in the sample well calculated from standard curve (nmol); Sv = sample volume added in the sample wells (μL) and D = sample dilution factor.

### 2.9. Statistical Analysis

The statistical analyses for this study were conducted utilizing GraphPad Prism 9.3.0 software. The experiments were performed two times with three technical replicates for each treatment. A two-way ANOVA was performed on three distinct group samples, yielding statistically significant results (*p* < 0.05).

## 3. Results

### 3.1. Cytotoxic Effect of Honey on SSCs

**Figure 3:**
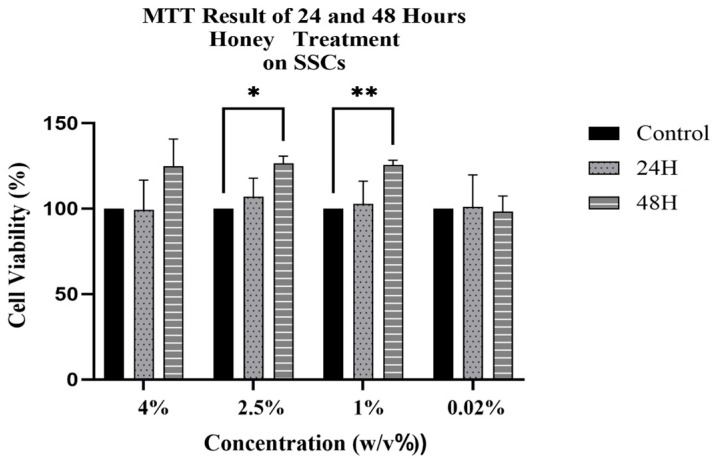
MTT results of honey-treated (H) SSCs at 24 and 48 h. Data are compared with the untreated control group (set at 100). Data are expressed as mean ± SD (*n* = 6) referring to the control. The experiments were performed two times with three technical replicates for each treatment. Statistical analysis was performed using Two-way ANOVA in GraphPad. Significance levels: * *p* < 0.05, ** *p* < 0.01.

We observed no cytotoxic effect on stem cells after treatment with honey for 24 and 48 h, for each concentration analyzed, with a general increase in cell viability (Figure 3). The 1% concentration of honey significantly upregulated stem cell metabolic activity. Therefore, subsequent experiments were performed using this 1% concentration of Honey.

### 3.2. Cytotoxic Effect of Honey on HFF1

**Figure 4:**
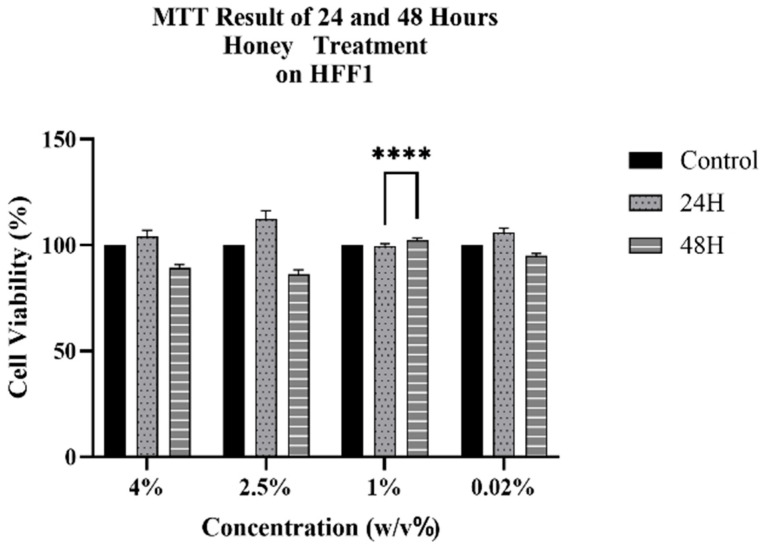
MTT results of honey-treated (H) HFF1 cells at 24 and 48 h. Data are compared with the untreated control group (set at 100). Data are expressed as mean ± SD (*n* = 6) referring to the control. The experiments were performed two times with three technical replicates for each treatment. Statistical analysis was performed using Two-way ANOVA in GraphPad. Significance levels: **** *p* < 0.0001.

We observed no cytotoxic effect on HFF1 after treatment with honey for 24 and 48 h, for each concentration analyzed, with a general increase in cell viability (Figure 4). According to the MTT results of stem cells, the selected 1% concentration showed a statistically significant increase in HFF1 viability and was therefore selected for subsequent experiments.

### 3.3. Gene Expression Analysis in SSCs and HFF1 Cells

#### 3.3.1. Gene Expression Analysis of Stem Cell and Cell Cycle Related Genes

**Figure 5:**
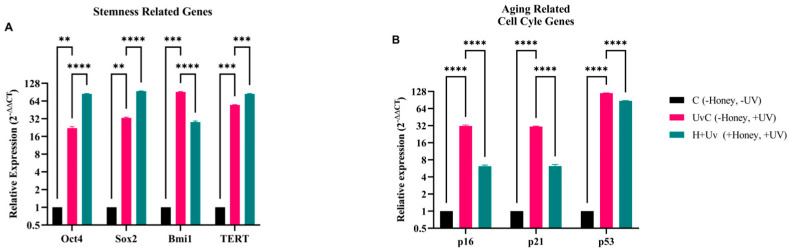
Gene expression analysis in SSCs. (**A**) Expression of Oct4, Sox2, Bmi1, and TERT. (**B**) Expression of p16, p21, and p53. mRNA levels of UvC (pink) and H + Uv (green) are expressed as 2^−∆∆Ct^ relative to the control (C = 1). Data are expressed as mean ± SD (*n* = 6) referring to the control. The experiments were performed two times with three technical replicates for each treatment. Statistical analysis was performed using Two-way ANOVA in GraphPad. Significance levels: ** *p* < 0.01, *** *p* < 0.001, and **** *p* < 0.0001.

RT-qPCR results show a statistically significantly increase (*p* < 0.05) in the expression of stem cell-related genes Oct4, Sox2 and TERT in the H + Uv SSCs, as compared to the UvC, while Bmi1 expression was significantly decreased (Figure 5). The expression of stem cell-associated genes was significantly increased in the UvC as compared to the C. The UvC showed a significantly increased in the expression of all aging and cell cycle-related genes as compared to the C. The expressions of p16, p21, and p53, which are aging and cell cycle-related genes, were significantly reduced in the H + Uv than in the UvC.

#### 3.3.2. Gene Expression Analysis of Wnt Signaling Pathway in Stem Cells

**Figure 6:**
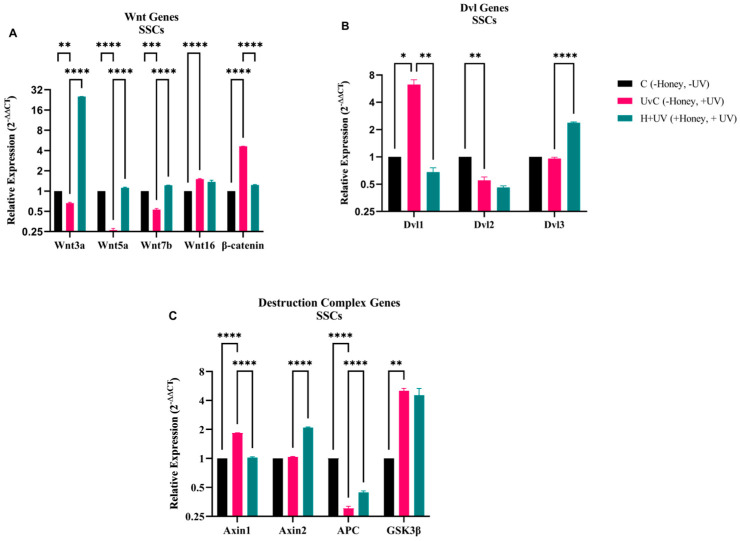
Gene expression analysis of Wnt signaling pathway in SSCs. (**A**) Wnt3a, Wnt5a, Wnt7b, Wnt16, and β-catenin. (**B**) Dvl1, Dvl2, and Dvl3. (**C**) Axin1, Axin2, APC, and GSK3β. mRNA levels of UvC (pink) and H + Uv (green) are expressed as 2^−∆∆Ct^ relative to the control (C = 1). Data are expressed as mean ± SD (*n* = 6) referring to the control. The experiments were performed two times with three technical replicates for each treatment. Statistical analysis was performed using Two-way ANOVA in GraphPad. Significance levels: * *p* < 0.05, ** *p* < 0.01, *** *p* < 0.001, and **** *p* < 0.0001.

The RT-qPCR results revealed a significant increase in Wnt3a, Wnt5a, and Wnt7b expression in the H + Uv as compared to the UvC (Figure 6). Wnt16 expression showed no significant change. A statistically significant decrease in β-catenin expression was observed in the H + Uv as compared to the UvC. The expression of other Wnt genes except Wnt16 and β-catenin was significantly decreased in the UvC as compared to the C. In the UvC, Dvl1 gene expression increased significantly compared to the C, while Dvl2 expression was decreased. The H + Uv SSCs exhibited statistically significantly (*p* < 0.05) lower levels of Dvl1 as compared to UvC. In contrast to Dvl1, Dvl3 showed a statistically significant increase in H + Uv stem cells as compared to the UvC. We observed no significant differences between the UvC and H + Uv SSCs for Dvl2. Concerning DCs, the H + Uv showed a significant decrease in Axin1 expression as compared to the UvC, while they also showed a similar decrease in GSK3 expression, albeit not statistically significant. Compared to the UvC, the H + Uv showed significantly increased Axin2 and APC expression. The UvC showed significantly increased expression of Axin and GSK3, while suppressing the expression of APC.

#### 3.3.3. Gene Expression Analysis of Extracellular Matrix and Stress-Related Genes in HFF1

**Figure 7:**
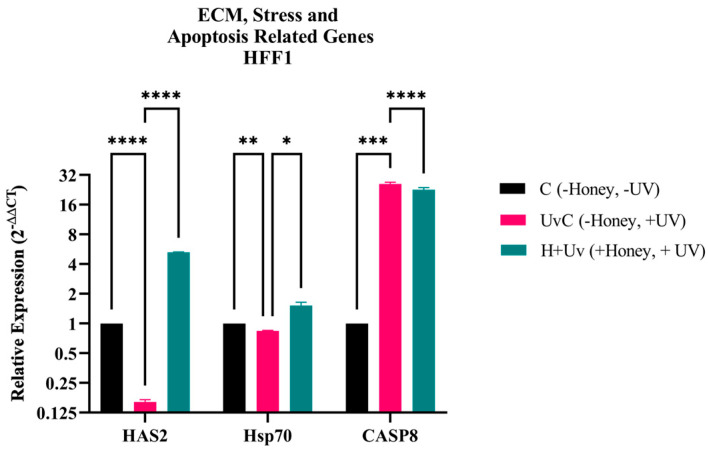
Gene expression analysis of HAS2, Hsp70, and CASP8 in HFF1. mRNA levels of UvC (pink) and H + Uv (green) are expressed 2^−∆∆Ct^ relative to the control (C = 1). Data are expressed as mean ± SD (*n* = 6) referring to the control. The experiments were performed two times with three technical replicates for each treatment. Statistical analysis was performed using Two-way ANOVA in GraphPad. Significance levels: * *p* < 0.05, ** *p* < 0.01, *** *p* < 0.001, and **** *p* < 0.0001.

RT-qPCR results revealed a significant suppression of HAS2 and Hsp70 expression in the UvC HFF1 as compared to the C (Figure 7). On the other hand, the expression of HAS2 and Hsp70 genes increased significantly (*p* < 0.005) in the H + Uv HFF1 as compared to the UvC. The UvC showed a significantly higher CASP8 expression as compared to the C, while the H + Uv showed a significant decrease in its expression as compared to the UvC.

#### 3.3.4. Gene Expression Analysis of Wnt Signaling Pathway in HFF1

**Figure 8:**
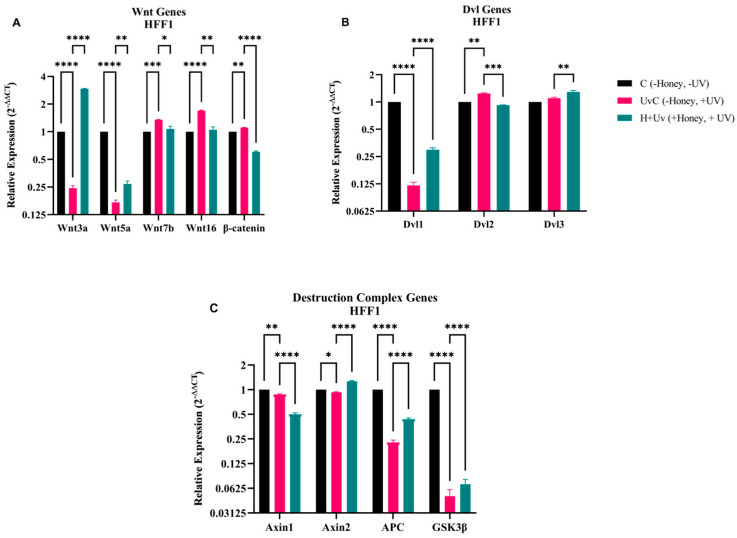
Gene expression analysis of Wnt signaling pathway in HFF1. (**A**) Wnt3a, Wnt5a, Wnt7b, Wnt16, and β-catenin. (**B**) Dvl1, Dvl2, and Dvl3. (**C**) Axin1, Axin2, APC, and GSK3β. mRNA levels of UvC (pink) and H + Uv (green) are expressed as 2^−∆∆Ct^ relative to the control (C = 1). Data are expressed as mean ± SD (*n* = 6) referring to the control. The experiments were performed two times with three technical replicates for each treatment. Statistical analysis was performed using Two-way ANOVA in GraphPad. Significance levels: * *p* < 0.05, ** *p* < 0.01, *** *p* < 0.001, and **** *p* < 0.0001.

The RT-qPCR results showed that Wnt3a and Wnt5a expression were significantly higher in the H + Uv as compared to the UvC (Figure 8). On the other hand, Wnt7b, Wnt16, and β-catenin expression were lower in HFF1 cells. The UvC showed a significantly increased in β-catenin, Wnt7b, and Wnt16 expression as compared to the C, while suppressing Wnt3a and Wnt5a expression. In the UvC, Dvl1 expressions in HFF1 cells decreased significantly as compared to the C, while Dvl2 expression was increased. In the H + Uv, Dvl1 and Dvl3 expressions significantly increased, while Dvl2 expressions significantly decreased as compared to the UvC. In the UvC, the expression of degradation complex genes decreased significantly as compared to the C. In the H + Uv, Axin2, APC, and GSK3β expressions were significantly increased as compared to the UvC. In contrast to other genes, Axin1 expression decreased in the H + Uv as compared to the UvC.

### 3.4. Honey Phytochemical Characterization by UHPLC-Orbitrap^®^-HRMS

The UHPLC-Orbitrap^®^-HRMS analysis revealed that the examined honey samples are particularly rich in phenolic acids and flavonoids. Among phenolic acids, 4-hydroxybenzoic acid (879.35–1075.68 ng/g), syringic acid (216.90–536.78 ng/g), and caffeic acid (290.87–312.20 ng/g) were found at relatively high concentrations. Regarding flavonoids, acacetin (19,292.94 ng/g), pinocembrin (479.94 ng/g), and chrysin (441.12 ng/g) were the most abundant. The presence of these compounds highlights their major contribution to the antioxidant potential of the honey [32,33,34,35,36,37,38] (Table 3).

The honey sample was re-analyzed in this study to confirm the reproducibility and reliability of the previously reported data. Certain compounds appeared in one analysis but not in the other or were present at varying concentration levels. Such discrepancies may result from differences in sample preparation, variations in instrument sensitivity, or natural changes in the honey matrix over time. Nevertheless, the overall phytochemical profile remained consistent across both analyses, supporting the reliability of the findings. For certain key phenolic compounds, including acacetin, quantitative confirmation was limited in the present analysis due to the unavailability of analytical standards; therefore, data from the previous analysis of the same honey sample were included for reference. Although quantitative differences were observed between the two analyses, their potential biological impact was not directly assessed and did not alter the interpretation of the results within the scope of the present study.

### 3.5. Antioxidant Activity in SSCs

**Figure 9:**
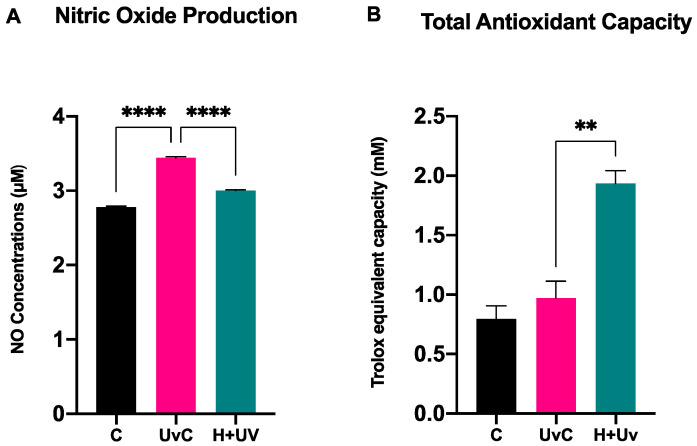
(**A**) Analysis of NO concentration and (**B**) Total Antioxidant Capacity in SSCs after 24 h. Analyses were performed using the GraphPad program ANOVA method. Data are expressed as mean ± SD (*n* = 6) referring to the control. The experiments were performed two times with three technical replicates for each treatment. Significance levels: ** *p* < 0.01 and **** *p* < 0.0001.

The concentration of NO (Figure 9A) and TAC (Figure 9B) were evaluated in SSCs cultured in the above-described conditions. The results showed reduced NO levels in (H + Uv) SSCs as compared to UvC, suggesting that the pretreatment modulated the enzymatic synthesis of NO, enhancing the cellular antioxidant capacity. These findings were confirmed by the TAC assay, which showed a significant increase in total antioxidant capacity in H + Uv SSCs as compared to UvC, improving the cellular response to oxidative stress induced by UV exposure.

## 4. Discussion

Skin integrity is maintained through a complex interaction between the regenerative capability of resident stem cells and the structural support provided by the dermal extracellular matrix (ECM). Ultraviolet (UV) radiation is a key external stress factor that accelerates skin aging by inducing DNA damage and oxidative stress and disrupting the stem cell niche [4]. In this study, the protective role of Eastern Anatolian multifloral honey was investigated using a dynamic bioreactor model, which provides a physiologically more relevant environment compared to static cultures.

In recent years, natural chemicals, in particular polyphenol-rich substances like honey, have been largely exploited for the prevention of UV-induced photo-damage. Honey is a complex solution comprising inverted sugars, phenolic acids, flavonoids, and many bioactive components, exhibiting potent antioxidant and anti-inflammatory activities [39,40]. Previous studies showed that honey-derived phytochemicals can modulate fundamental molecular pathways of aging [7,8,9,10,11]. Compounds as acacetin, pinocembrin, chrysin, syringic acid, and caffeic acid have been reported to activate stem cell–related signals (Oct4/Sox2, Wnt/β-catenin), stimulate longevity-associated enzymes (sirtuins), and alleviate oxidative stress and DNA damage. In parallel, they downregulate senescence-associated effectors, including p16, p21, and p53, thereby linking their skin-protective properties to broader anti-aging mechanisms [10,11]. In addition to these generalized anti-aging processes (Table 4), honey phytochemicals also induce robust protective actions in skin cells (Table 5). Specifically, they inhibit collagen breakdown induced by UV, activate intracellular antioxidant defense (e.g., SIRT3, Nrf2), repress inflammatory mediators (NF-κB, NLRP3, MMPs), and induce collagen formation and growth factor signaling, overall providing anti-aging and regenerative skin endpoints [41]. This study assessed the protective effects of honey on SSCs, and fibro-blast cells subjected to UV exposure, using a dynamic culture technique that simulates in vivo settings.

Tualang honey, known for its antioxidant and anti-inflammatory properties, has been shown to inhibit UV-induced DNA damage in skin cells and suppress p65 [42], an important factor in cyclin E expression [43]. In the present manuscript, two key cell populations representing the skin’s regenerative (SSCs) and structural (HFF1 fibroblasts) integrity were used, in order to further characterize UV-induced aging responses and the protective role of honey. In SSCs, pre-treatment with honey significantly increased the expression of key stemness genes, as Oct4, Sox2, and TERT, which are essential for pluripotency and self-renewal. Simultaneously, honey directly intervened in the molecular pathways of cellular aging by suppressing the activation of UV-induced senescence markers p16, p21, and p53. Although the roles of the Bmi1 and Wnt signaling pathways in stem cell protection and aging have been extensively described in the literature [21,22], the findings obtained in this study indicate that honey modulates these pathways in a context-specific manner, supporting the preservation of stem cell function rather than widespread proliferative activation under stressing conditions.

Analysis of components of the Wnt signaling pathway supports this interpretation. UV exposure disrupts the balance between Wnt ligands and β-catenin (Figure 6) [44,45,46], while honey application was associated with a partial restoration of the expression of some Wnt ligands, primarily Wnt3a, and a limitation of β-catenin accumulation. This modulation is consistent with the need to preserve both regeneration and genomic stability in aging tissues.

In contrast, in HFF1 fibroblasts UV exposure normally leads to ECM degeneration and fibroblast apoptosis [47]. This was confirmed by decreased HAS2 and Hsp70 and increased CASP8 in our UV control group (Figure 7). Pre-treatment with honey effectively reversed these effects, increasing HAS2 expression, indicating increased hyaluronan production, and elevating Hsp70 levels, which support protein homeostasis and DNA repair [23]. The suppression of CASP8 indicates a decrease in external apoptotic signals and preservation of the dermal framework (Figure 7).

The use of the dynamic system has enabled the simultaneous evaluation of these cell-type-specific responses within the same environment. A continuous flow rate of 0.1 mL/min supported the exchange of culture medium and potential signaling molecules between the HaCaT (epidermal barrier simulation), SSC, and HFF1 chambers. The observed holistic “anti-aging” effect (preservation of regenerative capacity in SSC and maintenance of supportive niche in HFF1) may be related to the coordinated cellular responses provided by the dynamic model.

The biological activity of honey may be related to its unique phytochemical profile, which contains high concentrations of acacetin (19,292.94 ng/g), pinosembrin, and caffeic acid (Table 3). And also high proline content (943.8 mg/kg). Within this context, the “component–target–phenotype” relationship observed in the study is particularly noteworthy in some components belonging to the Wnt signaling pathway. UV radiation was observed to negatively affect Wnt pathway regulation by reducing the expression of ligands, as Wnt3a and Wnt5a and disrupting signal balance (Figure 6 and Figure 8). After honey application, we observed that the expression of these ligands was partially preserved and that a tendency towards restoring pathway balance along with changes in the expression of regulatory components such as Axin2 and APC (Figure 6), could be observed. This controlled modulation in the Wnt/β-catenin axis suggests that honey-derived phenolic compounds may contribute to alleviating cellular aging responses and supporting renewal processes. However, attributing this effect to a single compound or a single pathway mechanism is not possible with the current data. Additionally, honey significantly modulated TAC levels and reduced NO production (Figure 9), reinforcing the antioxidant defense.

These findings indicate that the effect of honey on the activation of Wnt signaling pathway is finely tuned according to the type of damage and biological context. While the therapeutic potential of the Wnt signaling pathway is well established in the context of acute tissue repair [44,45,46,48], its role in the skin aging process requires more delicate and homeostatic regulation [17]. Recent studies on bioactive peptides, as RL-QN15 have shown that strong activation of the FZD8–β-catenin axis supports wound regeneration [49]. Similarly, the integrin agonist FZ1 has been reported to contribute to healing processes by enhancing angiogenesis and tissue repair [50]. However, in aging associated chronic UV exposure, excessive or uncontrolled Wnt activation may adversely affect stem cell functions and increase the risk of abnormal proliferation [13,20,51].

Our results suggest that the effect of multifloral honey can be considered as a regulatory modulation rather than pure Wnt activation. Unlike the acute activation observed in wound healing models, pretreatment with honey created a response profile characterized by partial preservation of Wnt ligand (Wnt3a, Wnt5a) expression, along with increased levels of degradation complex components, as Axin2 and APC and balanced β-catenin levels.

**Table 4:** Some polyphenols’ effects.

Compound	Main Effects	Target Pathways/Genes
Acacetin	Anti-senescence, telomere protection, UVA protection	↓ p53, ↓ p21, ↑ Sirt1/Sirt6, ↑ SIRT3, TGF-β/Smad3, mitochondrial function [10,11]
Pinocembrin	Antioxidant, wound healing, stemness support	↑ Wnt/β-catenin (via Fzd1), ↓ NF-κB [7,8]
Chrysin	Context-dependent: protects normal stem cells, induces senescence/apoptosis in cancer	↑ p53, ↑ p21, ↑ p16; ↓ ROS; maintains HSC self-renewal [52,53]
4-Hydroxybenzoic acid (4-HBA)	Epigenetic modulator	HDAC6 inhibition → ↑ p53 activity, ↑ HIPK2/p53 pathway [54]
Caffeic acid	Anti-fibrotic, antioxidant; Anti-ionizing radiation	↓ Nox4/ROS, ↓ α-SMA, ↓ MMP-1; ↑ Nrf2 [55,56]

↑: Increase, ↓: Decrease.

**Table 5:** In vivo and in vitro effect of some polyphenols on skin.

Compound	Model/Cell Type	Dose/Treatment	Mechanism of Action/Main Findings
Acacetin	UVA-irradiated mice and human dermal fibroblasts (HDF), HaCaT keratinocytes,	40–80 mg/kg topical (mice), 5–20 μg/mL (cells)	Prevented photoaging, preserving collagen and decreasing wrinkles and inflammation [10,57]; ↓ MAPK/AP-1 (JNK/c-Jun) and MMP-1/3, preserves SIRT3, ↓ ROS, ↑ TGF-β/Smad3
Pinocembrin	Human keloid fibroblasts, mouse dermal fibroblasts, bleomycin-induced skin fibrosis (mice). HaCaT cell line	20–80 µM (cells); intradermal in mice. 0.5–500 µM	Suppressed keratinocytes and fibroblast proliferation/migration, reducing fibrosis and scar formation [9,58]; ↓ TGF-β1/Smad, ↓ α-SMA, collagen I and fibronectin. ↑ MAPK and PI3K/Akt signaling pathways.
Chrysin	Human dermal fibroblasts, B16 murine melanoma cells, amiodarone-induced skin injury (rats)	6.25–25 μM (cells); 10–40 mg/mL topical (rats)	Improved collagen production, reduced oxidative stress, accelerated wound closure and re-epithelialization [59,60]; ↓ degradation of collagen I synthesis, SA-β-Gal and MMP1 activity, ↓ IL-6/TNF-α, ↑ bFGF
4-Hydroxybenzoic acid	UV-damaged skin cells (mainly from plant extracts)	Not specified	Reduced inflammation, supported repair and antioxidant activity, protecting against photoaging [61]; ↓ UV-induced pathways
Syringic acid	Diabetic wound model (rats), HaCaT keratinocytes (C. acnes-induced inflammation); L-929 fibroblasts	2.5–5% ointment (rats); 0.4–32 µM (cells); 1–200 μM (cells)	Accelerated wound healing improving collagen and angiogenesis; reduced oxidative/inflammatory markers and ROS, preventing lipid peroxidation [62,63,64]; ↓ NF-κB/NLRP3, ↑ PPARγ/Nrf2, ↓ MMPs, raises TIMPs, boosts TGF-β1 and VEGF;
Caffeic acid	Mouse skin incision model, NIH-3T3 fibroblasts, RAW 264.7 macrophages, MSCs; HDFa, albino mouse	10 mg/kg oral (mice), 30–100 µM (cells); 10–100 μM; 40 μM (cells), 15 mg kg.b.wt (mouse)	Counteract photoaging, faster wound healing, reducing oxidative damages and inflammation, enhancing collagen deposition, and improving MSC survival under hypoxia [41,65,66]; ↓ MPO/ROS, ↓ PLA_2_ and PGE_2_, boosts collagen; ↓ PI3K and AKT and ↑ PTEN

↑: Increase, ↓: Decrease.

## 5. Conclusions

In conclusion, our findings demonstrate that honey treatment significantly upholds cellular homeostasis by upregulating pluripotency-associated genes, Oct4 and Sox2, crucial regulators of regenerative potential. Furthermore, the activation of the Wnt3a signaling pathway plays a pivotal role in mediating tissue repair, while Hsp70 increased expression, provides a robust defense against stress. These integrated pathways collectively represent the mechanistic basis for honey’s cytoprotective properties, paving the way for its application in regenerative medicine.

### Limitations

A significant limitation of this study is that, although the dynamic system allows a paracrine crosstalk through continuous medium flow, intercellular communication between the different cell types (SSCs and HFF1) was not specifically investigated or quantified. Our current findings are focused on the cell-type-specific molecular responses within a shared dynamic environment. Future studies utilizing secretome analysis or metabolic profiling are necessary to fully elucidate the synergistic effects and signaling pathways facilitated by this dynamic co-culture setup. While cellular senescence was validated through the modulation of key molecular hallmarks (p16, p21, and p53), functional assays, as SA-β-gal staining were not performed. We acknowledge this as a limitation; therefore, future research will include these phenotypic assessments to complement the molecular protective effects observed in our current dynamic model.

## Figures and Tables

**Table 1:** HFF1 and SSCs culturing condition in Bioreactor.

Experimental Grups	Honey Treatment	UV Damage
Control (C)	No	No
UV Control (UvC)	No	Yes
UV Honey (H + Uv)	Yes	Yes

**Table 2:** Primer list [30,31].

	Forward Primer	Reverse Primer
β-Catenin	5′-CTTACACCCACCATCCCACT-3′	5′-CCTCCACAAATTGCTGCTGT-3′
Dvl1	5′-CCACCCTGAACCTCAACAGT-3′	5′-CCTTCACTCTGCTGACTCCC-3′
Dvl2	5′-GCCTATCCAGGTTCCTCCTC-3′	5′-AGAGCCAGTCAACCACATCC-3′
Dvl3	5′-TTCCATCCCTGACACAGAGC-3′	5′-TCCGTGAAGCCTTCCACATT-3′
GAPDH	5′-GCACCACCAACTGCTTA-3′	5′-AGTAGAGGCAGGGATGAT-3′
Wnt3a	5′-ACCTGAAGGCAGGGCTCCTC- 3′	5′-GCGTACGTGAAGGCCGTCTC-3′
Wnt5a	5′-TTTCTCCTTCGCCCAGGTTG-3′	5′-GCGTACGTGAAGGCCGTCTC-3′
Wnt7b	5′-AGAAGACCGTCTTCGGGCAAGA-3′	5′-AGTTGCTCAGGTTCCCTTGGCT-3′
Wnt16	5′-CGCTGAACAGCCGCCAGAAG-3′	5′-ACAGCACAGGAGCCGGAAAC-3′
GSK3β	5-AATGAACCCAAACTACACAGAATTTAAA-3	5′-CAATTGCCTCCGGTGGAGT-3′
APC	5′-GCCCCTGACCAAAAAGGAAC-3′	5′-TGGCAGCAACAGTCCCACTA-3′
Axin1	5′-AGCCGTGTCGGACATGGA-3′	5′-AAGTAGTACGCCACAACGATGCT-3′
Axin2	5′-TGTGAGGTCCACGGAAACTG-3′	5′-CGTCAGCGCATCACTGGATA-3′
Oct4	5′-CTCACCCTGGGGGTTCTATT-3′	5′-CTCCAGGTTGCCTCTCACTC-3′
Sox2	5′-GCACATGAACGGCTGGAGCAACG-3′	5′-TGCTGCGAGTAGGACATGCTGTAGG-3′
p16	5′-CATGAGTGTGGATCCAGCTTG-3′	5′-CCTGAATAAGCAGATCCATGG-3′
p19	5′-CAACGCACCGAATAGTTACGG-3′	5′-AACTTCGTCCTCCAGAGTCGC-3′
p21	5′-GCCTTCGGCTGACTGGCTGG-3′	5′-TCGTCCTCCAGAGTCGCCCG-3′
p53	5′-CAAAGGCCCGCTCTACATCTT-3′	5′-AGGAACCTCTCATTCACCCGA-3′
Bmi1	5′-TGGCCTTGAAACCACCTTTT-3′	5′-AACTACCAACCCACCAGCCAA-3′
TERT	5′-GCCAACAGCCCAGCAGGAGG-3′	5′-TTGGTGGTTACCGCTGGGGC-3′
HAS2	5′-GACGTGGAAGATGAGCGTG-3′	5′-GACGACGTACACACTCATC-3′
CASP8	5′-TCCACGAAAAGGGTCCCGGTGA-3′	5′-TCGTCCCAGTGCTCTGAAGGCT-3′
Hsp70	5′-AGGAGGAGATGGAAAGGGAACTT-3′	5′-ACCTCAATTCTGATCTGCTCACTTCT-3′

**Table 3:** Analytical parameters of the UHPLC-Orbitrap^®^-HRMS method, UHPLC-Orbitrap -HRMS analysis was performed to determine up to 81 phytochemical profiles of the honey samples.

Target Compounds	Calculated Concentration (1)	Calculated Concentration (2)
Acacetin (5,7-Dihydroxy-2-(4-methoxyphenyl)-4H-chromen-4-one)	19,292.94	NS
Naringenin	1169.75	1702.24
4-Hydroxybenzoic acid	1075.68	879.35
Quinic acid	414.53	NF
Salicylic acid	354.04	274.83
Caffeic acid	312.2	290.87
Epigallocatechin gallate	NF	307.49
Quercetin	266.28	314.64
Isorhamnetin (Quercetin 3′-methyl ether)	266.12	334.74
2,4-dihydroxybenzoic acid (beta-Resorcylic acid)	240.68	NF
Nicotiflorin (Kaempferol 3-rutinoside, Kaempferol 3-O-β –rutinoside)	235.95	188.84
Syringic acid	216.9	536.78
Coumaric acid (trans-3-Hydroxycinnamic acid)	216.83	219.25
Galangin (3,5,7-Trihydroxy-2-phenyl-4H-chromen-4-one)	NF	202.15
Ferulic acid	NF	191.4
Rutin hydrate M-OH2	138.06	NF
3-(4-Hydroxyphenyl) propionic acid	133.83	NF
Vanillic acid	120.88	NF
Protocatechuic acid (3,4-Dihydroxybenzoic acid)	113.03	NF
Benzoic acid	NS	71.81
Narcissin (Narcissoside, Isorhamnetin 3-rutinoside)	66.57	79.48
Apigenin (5,7-Dihydroxy-2-(4-hydroxyphenyl)-4H-chromen-4-one)	52.18	92.87
Eriodictyol (3,4,5,7-Tetrahydroxyflavanone)	42.34	NF
Ellagic acid	41.34	NF

Concentration 1 = previous study [26]. Concentration 2 = this study. NF: Not founded. NS: Not in the standards.

## Data Availability

The original contributions presented in this study are included in the article. Further inquiries can be directed to the corresponding authors.

## Abbreviations

The following abbreviations are used in this manuscript:

α-SMA	Alpha Smooth Muscle Actin
APC	Adenomatous Polyposis Coli
AKT	Protein Kinase B
ATCC	American Type Culture Collection
ATP	Adenosine Triphosphate
Axin1	Axis Inhibition Protein 1
Axin2	Axis Inhibition Protein 2
bFGF	Basic Fibroblast Growth Factor
Bmi1	B lymphoma Mo-MLV insertion region 1
β-catenin	Beta catenin
CASP8	Caspase 8
CDKN1A	Cyclin Dependent Kinase Inhibitor 1A
CDKN2A	Cyclin Dependent Kinase Inhibitor 2A
CNR	Consiglio Nazionale delle Ricerche
Ct	Cycle Threshold
DMSO	Dimethyl Sulfoxide
DMEM	Dulbecco’s Modified Eagle Medium
DNA	Deoxyribonucleic Acid
DVL	Dishevelled Protein Family (Dvl1, Dvl2, Dvl3)
ECM	Extracellular Matrix
FBS	Fetal Bovine Serum
GAPDH	Glyceraldehyde 3 phosphate dehydrogenase
GSK3β	Glycogen Synthase Kinase 3 Beta
HaCaT	Human Keratinocyte Cell Line
HAS2	Hyaluronan Synthase 2
HDF	Human Dermal Fibroblast
HFF1	Human Foreskin Fibroblast 1
HIPK2	Homeodomain Interacting Protein Kinase 2
HRMS	High Resolution Mass Spectrometry
Hsp70	Heat Shock Protein 70
HSC	Hematopoietic Stem Cell
IL-1β	Interleukin 1 Beta
MAPK	Mitogen Activated Protein Kinase
MMP	Matrix Metalloproteinases (MMP-1, MMP-3)
MSCs	Mesenchymal Stem Cells
MTT	MTT Cell Viability Assay
NLRP3	NLR Family Pyrin Domain Containing 3
NF-κB	Nuclear Factor Kappa B
Nrf2	Nuclear Factor Erythroid 2 related Factor 2
OD	Optical Density
PBS	Phosphate-Buffered Saline
PCR	Polymerase Chain Reaction
PI3K	Phosphoinositide 3 Kinase
PPARγ	Peroxisome Proliferator Activated Receptor Gamma
p16	Cyclin Dependent Kinase Inhibitor 2A
p21	Cyclin Dependent Kinase Inhibitor 1A
p53	Tumor Protein 53
PTEN	Phosphatase and Tensin Homolog
RNA	Ribonucleic Acid
ROS	Reactive Oxygen Species
RT-qPCR	Real Time Quantitative Polymerase Chain Reaction
SIRT	Sirtuin Protein Family (SIRT1, SIRT3, SIRT6)
SSCs	Skin Stem Cells
TGF-β1	Transforming Growth Factor Beta 1
TERT	Telomerase Reverse Transcriptase
UV	Ultraviolet
UVB	Ultraviolet B
UVC	Ultraviolet C
VEGF	Vascular Endothelial Growth Factor
WNT	Wingless Type Signaling Proteins (Wnt3a, Wnt5a, Wnt7b, Wnt16)
